# Effects of Breathing Exercises on Neck Pain Management: A Systematic Review with Meta-Analysis

**DOI:** 10.3390/jcm14030709

**Published:** 2025-01-22

**Authors:** Antonello Cefalì, Davide Santini, Giovanni Lopez, Filippo Maselli, Giacomo Rossettini, Mauro Crestani, Graziana Lullo, Ian Young, James Dunning, Raphael Martins de Abreu, Firas Mourad

**Affiliations:** 1 Department of Health, LUNEX University of Applied Sciences, 4671 Differdange, Luxembourg; 2 Luxembourg Health & Sport Sciences Research Institute A.s.b.l., 4671 Differdange, Luxembourg; 3 Department of Human Neurosciencies, Sapienza University of Rome, 00185 Rome, Italy; 4 Sovrintendenza Sanitaria Regionale Puglia INAIL, 70126 Bari, Italy; 5 School of Physiotherapy, University of Verona, 37134 Verona, Italy; 6 Department of Physiotherapy, Faculty of Sport Sciences, Universidad Europea de Madrid, 28670 Villaviciosa de Odón, Spain; 7 Department of Neurosciences, Biomedicine and Movement Sciences, University of Verona, 37134 Verona, Italy; 8 American Academy of Manipulative Therapy Fellowship in Orthopaedic Manual Physical Therapy, Montgomery, AL 36104, USA; 9 Tybee Wellness & Osteopractic, Tybee Island, GA 31328, USA; 10 Montgomery Osteopractic Physical Therapy & Acupuncture Clinic, Montgomery, AL 36106, USA

**Keywords:** neck pain, breathing exercise, respiration, disability, pulmonary function

## Abstract

**Background:** Given the relationship between reduced pulmonary and respiratory muscle function in neck pain, incorporating breathing exercises into neck pain management may be beneficial. **Purpose**: The purpose of this study was to investigate the benefits of breathing exercises for treating neck pain. **Methods**: We searched PubMed (MEDLINE), PEDro, CINAHL, Scopus, and EMBASE databases, up to the 28 of February 2024. Randomized controlled trials evaluating the impact of breathing exercises on reducing pain and disability in both persistent and recent neck pain were selected. A meta-analysis was conducted for each outcome of interest; however, if quantitative methods were not possible, a qualitative synthesis approach was used. The risk of bias was assessed using the Cochrane RoB 2.0 Tool (version 22 August 2019). We used the GRADE approach to judge the certainty of the evidence. **Results**: Five studies were included. Meta-analysis showed a statistically significant reduction in pain (standardized mean difference (SMD), −10.16; 95% CI: −14.82, −5.50) and disability (SMD, −0.80; 95% CI: −1.49, −0.11), in favor of breathing exercises. Qualitative synthesis for pulmonary functional parameters resulted in a statistically significant improvement for FVC, MIP, MEP, and MVV, in favor of breathing exercises. **Conclusions**: Breathing exercises showed significant short-term effects in reducing pain and disability for persistent neck pain. They also provided benefits for functional respiratory parameters. However, the evidence certainty is low.

## 1. Introduction

Neck pain is a widespread and debilitating musculoskeletal condition. It results in substantial self-reported pain and disability, and it imposes a significant burden on personal well-being and worldwide healthcare systems [1]. Economic consequences include the cost of healthcare, reduced work productivity, work absenteeism and insurance [2]. Globally, the age-standardized prevalence of neck pain is estimated to be 2450 cases per 100,000 population, with an estimated rate of 244 per 100,000 age-standardized years lived with disability [1]. The number of cases is expected to rise of approximately 32.5% by 2050 [1].

The onset, course, and prognosis depend on multiple factors across biological, psychological, and social dimensions [3,4]. Based on the stage, neck pain may be classified as recent (0 to 3 months) or persistent (more than 3 months) [5]. Persistent neck pain is a complex biopsychosocial disorder with problematic physical and psychological symptoms [5]. Although the mechanisms are not fully understood, there is a reciprocal coupling between subjective and objective respiratory dysfunctions, mental and emotional health, and thus chronic pain [2,6]. Three recent systematic reviews [2,3,7] observed a significant reduction in respiratory muscle strength, along with decreased pulmonary function parameters (such as FEV (forced expiratory volume) 25–75 or the FEV1/FVC (forced vital capacity) ratio) in persistent neck pain patients but not in people suffering from persistent pain (without neck pain) [2,3,7].

Neck pain has been observed to alter the activation of neck muscles, increasing the recruitment of superficial cervical flexor muscles, and reducing the function (e.g., coordination and endurance) of deep cervical flexor muscles [2,7,8]. As cervical muscles contribute to breathing, these sensorimotor changes may also contribute to respiratory dysfunction [2,9]. Particularly, superficial neck muscles (e.g., scalene, the sternocleido-mastoid, and the trapezius) participate in inspiration [2,9]. The latter becomes particularly involved during periods of increased respiratory demand, such as physical exertion or respiratory distress. As accessory muscles of respiration, they contribute to elevate the ribcage and increase inspiratory volume [10,11]. Impairments in breathing accessory muscles can lead to respiratory dysfunctions. A correlation between persistent neck pain and respiratory dysfunction has been observed resulting in a reduced lung function [2,7]. Changes in respiratory function may also be related to the anatomical relationship be-tween the cervical and thoracic spine due to thoracic spine biomechanics alterations in patients with persistent neck pain [12]. 

Given the relationship between reduced pulmonary and respiratory muscle functions in persistent neck pain, incorporating breathing exercises into neck pain man-agreement may be beneficial [2,7,13]. Breathing exercises encompass various interventions, including diaphragmatic breathing, respiratory muscle training, deep breathing, balloon breathing, box breathing, and the active cycle of breathing techniques [14,15,16,17]. Diaphragmatic breathing, also known as belly or abdominal breathing, promotes efficient ventilation and reduces oxygen consumption during relaxed breathing [18]. Conversely, increased reliance on accessory muscles enhances the mechanical effort required for breathing, reducing ventilation efficiency, and may play a role to neck pain persistence [2,18]. Additionally, slow diaphragmatic breathing stimulates the vagus nerve, reducing peripheral inflammatory cytokines, lowering sympathetic tone, decreasing oxidative stress, modifying brain activation patterns related to pain, and regulating opioid effects [8]. Patients with persistent neck pain have observed possessing elevated levels of pro-inflammatory cytokines (i.e., IL-1β and TNFα), suggesting a relationship between an ongoing inflammatory process, diaphragmatic breathing dysfunction, and pain [19]. In addition, breathing training was found beneficial in short-term pain reduction and im-proved muscle activity of superficial neck muscles, cervical range of motion, and enhanced chest mobility in persistent neck pain [20]. Similar relationship has been observed in subjects suffering from low back pain. Muscles of the trunk perform both postural and respiratory functions, dysfunctions in one can affect the other [21,22]. Breathing rehabilitation in these subjects has been shown to reduce pain intensity, and to improve spirometry, respiratory function, and gas exchange [21].

Respiratory muscle training to address respiratory dysfunction in neck pain has been recommended in recent reviews [2,7]. However, these reviews did not investigate the effectiveness of respiratory muscle training or any respiratory intervention in people suffering from persistent neck pain [2,7]. To the best of the authors’ knowledge, no re-views have investigated the effects of breathing exercise alone in reducing pain and disability in patients with recent and persistent neck pain. Therefore, our systematic review aims to investigate the benefits of respiratory training and any other breathing exercise in the management of neck pain. We also aim to clarify the most common types and protocols of breathing exercises, guiding practitioners in prescribing this additional intervention for neck pain management.

## 2. Methods

This systematic review was conducted following the Cochrane Handbook for Systematic Reviews guidelines [23] and is reported in accordance with the updated Preferred Reporting Items for Systematic Reviews and Meta-Analyses (PRISMA) 2020 statement guidelines [24]. Additionally, the search strategies are documented according to PRISMA-S guidelines for reporting literature searches [25].

### 2.1. Protocol and Registration

The review protocol was registered with the International Prospective Register of Systematic Reviews (PROSPERO) on 23 March 2024 (registration number CRD42024518794). 

### 2.2. Search Methods and Strategy for Primary Studies

The searches were carried out from inception to 28 February 2024, on the following electronic databases: PubMed (MEDLINE), PEDro, CINAHL, Scopus, and EMBASE. Additionally, we searched the reference lists of the included articles, other systematic reviews [2,3,7], and in the relevant gray literature sources, such as Google Scholar. The search strategies are shown in Appendix A.

### 2.3. Eligibility Criteria

The scientific question and eligibility criteria were developed according to the PICOS framework as follows: Population (P): Studies including patients aged 18 and older with recent (less than 12 weeks) or persistent (more than 12 weeks) nonspecific neck pain or whiplash were included. Studies recruiting participants with serious pathologies such as cancer, inflammatory diseases, fractures, infections and myelopathy or specific diagnoses like radicular and neuropathic pain, radiculopathy, arthritis, osteoporosis, chronic obstructive pulmonary disease, dyspnea, and headache were excluded.

Intervention (I): We included any form of breathing exercises such as diaphragmatic breathing, respiratory muscle training, deep breathing, box breathing, balloon breathing and active cycle of breathing techniques [14,15,16,17].

Control (C): We included any non-invasive conservative interventions for nonspecific neck pain. This could encompass spinal manipulative therapy, exercises (e.g., deep cervical flexor strengthening), routine physiotherapy, sham interventions, and no intervention (including breathing exercise as an adjuvant to any other therapy).

Outcome Measures (O) and Follow-up: We included trials that considered at least one of the following outcomes: pain intensity measured by the numeric rating scale (NRS) or visual analog scale (VAS); disability measured by the Neck Disability Index (NDI) or Neck Bournemouth Questionnaire (NBQ); health related quality of life (HRQoL) assessed with generic-specific patient-reported questionnaires (e.g., Short-Form 12 (SF-12)). Pulmonary function parameters were also reported, including: FEV in the first second (FEV1), Peak Expiratory Flow (PEF), Vital Capacity (VC), FVC, Forced Expiratory flow between 25% and 75% of vital capacity (FEF25–75), Maximal Voluntary Ventilation (MVV) and the ratio FEV1/FVC. Moreover, the Strength of inspiratory and expiratory muscles were measured by the Maximal Inspiratory Pressure (MIP) and Maximal Expiratory Pressure (MEP), respectively.

Study design (S): We included only randomized controlled trials (RCTs) published in English.

### 2.4. Study Selection and Data Collection Process

The screening process was systematized using Rayyan https://help.rayyan.ai/hc/en-us/articles/4406419348369-What-is-the-version-of-Rayyan (accessed on 3 March 2024) [26]. Additionally, Zotero (version 6.0.37) was used to manage the bibliography. Screening of titles and abstracts was performed to identify potentially eligible records. Subsequently, a full-text assessment for eligibility was conducted, documenting reasons for exclusion. Data extraction was performed using a standardized extraction sheet, including the author and publication year, sample characteristics (including age, sex, and pain duration), intervention and control features, outcome measures, main results, and follow-up periods. The Template for Intervention Description and Replication (TIDieR) was used to ensure a comprehensive description of all interventions [27].

At any phase where full texts or data were unavailable—or to provide missing/additional data—authors of eligible studies were contacted via email twice, with a one-week interval between attempts. The selection and extraction phases were performed by two independent and blinded reviewers (A.C., D.S., Gr. Lu., M.C.). Any disagreement between the reviewers was resolved by consulting a third, independent reviewer (F.Mo.).

### 2.5. Risk of Bias in Individual Studies

The risk of bias of each outcome was assessed independently by two authors (A.C. and D.S.) using the Revised Cochrane risk-of-bias tool for randomized trials (RoB 2). The RoB graph was created using the RobVis visualization tool (version 22 August 2019). The following characteristics were assessed: methods of randomization, treatment allocation, blinding, completeness of outcome data, selective outcome reporting, similarity of groups’ baseline, and other sources of biases. The assessment was conducted at study and outcome levels. A consensus on disagreements between the reviewers was reached by consulting a third, independent reviewer (F.Mo.).

### 2.6. Data Synthesis

The analysis was structured as follows: for the primary analysis, we evaluated the effect of breathing exercises in comparison to any other conservative therapies, such as sham intervention and routine physiotherapy. In the secondary, analysis we verified the eventual differences between persistent and recent neck pain, if feasible.

### 2.7. Meta-Analysis

Changes in neck pain and NDI from baseline were analyzed in the meta-analysis by calculating the differences between post-intervention and pre-intervention values for both the breathing exercise and control groups. When the standard deviation (SD) of these changes was not reported, we estimated it using a correlation coefficient, following the guidelines from the Cochrane Handbook for Systematic Reviews of Interventions [28]. All meta-analyses were executed using Review Manager Software version 5.3, employing a random-effects model that incorporated heterogeneity into the model and was applied in both analyses. Statistical heterogeneity among the studies was assessed using Cochran’s Q test, with a *p*-value greater than 0.05 indicating statistical significance, and the I^2^ statistic, where a value greater than 50% was considered indicative of high heterogeneity. For continuous outcomes, we calculated the standardized mean difference (SMD), along with its corresponding 95% confidence interval (CI). Effectiveness was assessed based on clinical relevance and statistical significance. Clinical relevance was evaluated differently depending on the outcomes [29] and calculated by comparing the between pre- and post-treatment values to the minimal clinically important difference (MCID) thresholds. Statistical significance was determined by whether the 95% CI of the between-group effect excluded the null value.

### 2.8. Qualitative Synthesis

When meta-analysis was not possible, a qualitative synthesis approach was used. Data were extracted using a standardized form to extract study characteristics, populations, interventions, main outcomes, and findings. Results are reported through a narrative synthesis and a summary table that reports the key aspects of each study, including study interventions, quality assessment, and main findings.

### 2.9. Confidence in Cumulative Evidence

The quality of evidence and the strength of each outcome in the meta-analysis were evaluated using the Grading of Recommendations Assessment, Development, and Evaluation (GRADE) framework, following the recommendations outlined in chapter 14.2 of the Cochrane Handbook [30,31]. GRADEpro GDT (version 20 October 2021) was utilized to create the summary of findings tables, with one for each comparison [30]. These tables included the number of participants, assumed risk, treatment effect, and certainty of evidence for each comparison in the meta-analysis. The certainty of evidence may be reduced by factors such as risk of bias, imprecision, result inconsistency, indirect evidence, and publication bias. Conversely, factors like substantial effect sizes, dose–response relationships, and residual confounding can enhance certainty. Evidence certainty was categorized as high, moderate, low, or very low. Two reviewers (A.C. and D.S.) independently assessed the level of the evidence, with any disagreements resolved by consensus.

## 3. Results

**Figure 1:**
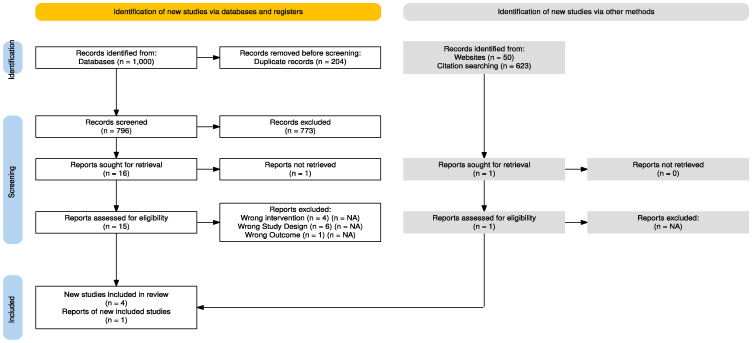
PRISMA flow diagram.

A total of 1000 records were identified from all databases. Two-hundred and four records were deleted as duplicates. Of the seven-hundred and ninety-six records initially screened by title and abstract, seven-hundred and seventy-three were considered unsuitable, and one record could not be retrieved. Fifteen full-text articles were screened, and four met the inclusion criteria and were included. One additional article was included from Google Scholar, with five final articles included. The process of study selection and the included trials are detailed in Figure 1 [32] and Appendix B, respectively. Excluded studies and the reason for exclusion are reported in Appendix C. One author [33] was contacted twice to obtain missing data, but no answer was received.

### 3.1. Included Studies

#### 3.1.1. Study Characteristics

A total of 228 patients (sample sizes ranging from 30 to 68), were included from five clinical trials [33,34,35,36,37]. All the included studies focus on populations with persistent neck pain [33,34,35,36,37] but none presents a population with recent neck pain. The average age of participants ranged from 20 to 50 years old. One study [35] included only female par-ticipants. The other four studies [33,34,36,37] included both males and females. Among these, three studies [33,34,37] had a higher proportion of males, while one study [36] did not specify the proportions. The characteristics of the included studies are reported in Table 1.

#### 3.1.2. Intervention Characteristics

The included studies used the following type of breathing exercises as intervention: diaphragmatic breathing exercise [33,34,35], balloon breathing [36] and respiratory muscle training [37]. Three studies [35,36,37] compared breathing exercises to a control group only. Control group therapies consisted of stretching, strengthening exercises, and passive modalities, such as Transcutaneous Electrical Nerve Stimulation (TENS) and Interferential Therapy (IFT). Two studies [33,34] compared breathing exercises to a control group plus sham breathing exercises.

The duration of the interventions varied from two to eight weeks, with different prescriptions across all the studies. Three studies [33,34,36] provided their interventions under supervision, while the other two [35,37] did not specify whether the interventions were supervised. Further details about the interventions of the included studies are reported in Appendix D.

### 3.2. Risk of Bias

**Figure 2:**
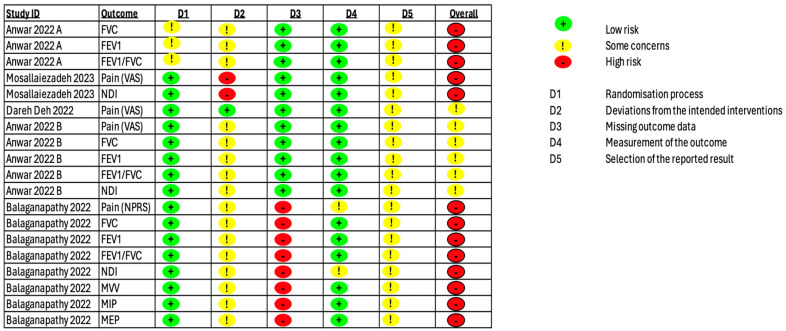
Risk-of-bias graph for the included studies.

Three trials (60%; n = 3/5) were considered to have an overall “high risk” of bias [34,35,37], while the two trials (40%; n = 2/5) had "some concerns" for all the investigated outcomes (Figure 2) [33,36].

#### 3.2.1. Effects of Interventions

Two separate meta-analyses were conducted for pain and disability against routine physiotherapy. Four trials (9 comparison) compared the effects on pain in the short term (2 to 8 weeks) [33,35,36,37], showing a statistically significant effect in favor of breathing exercises (SMD, −10.16; 95% CI: −14.82, −5.50; I2 = 98%; low evidence certainty). Three trials (6 comparisons) assessed the effect on NDI in the short term (2 to 8 weeks) [33,35,37] indicating a statistically significant effect in favor of breathing exercises (SMD, −0.80; 95% CI: −1.49, −0.11; I2 = 72%; low evidence certainty). 

**Figure 3:**
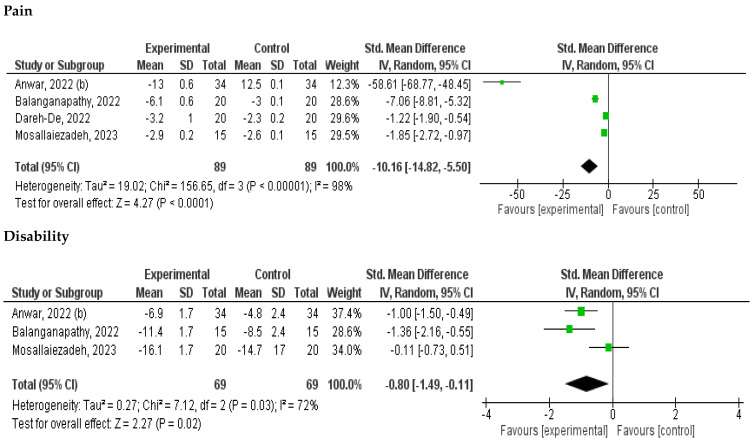
Forest plot of meta-analysis for pain and disability.

A secondary analysis was not feasible as all the included studies included patients with persistent neck pain. Results of meta-analyses are reported in Figure 3. Certainty of evidence is reported in Table 2.

#### 3.2.2. Qualitative Synthesis of Functional Respiratory Parameters

A meta-analysis of the functional respiratory parameters was not feasible due to the limited number of studies [34,37]. Three trials assessed the effects on FVC in the short-term (4 to 8 weeks) [33,34,37]. Of these, 2 studies [33,34] reported a statistically significant increase in FVC in the breathing exercise group compared to the control group, while one study 5 found no statistical difference in this variable in either of the groups studied. Additionally, two trials assessed the effects of breathing exercises on FEV1 at 8 weeks [33,34]; of these, only one [33] reported an increase of this index in favor of the breathing exercises group when compared to control group. 

Three trials assessed the impact of breathing exercises on FEV1/FVC at 8 weeks [33,34,37]. Only one [33] reported an increase in this ratio in favor of the breathing exercises group after training, when compared to the control group. Finally, only one study [37] investigated MIP, MEP, and MVV at 4 weeks, reporting increases in all outcomes in favor of the breathing exercises group. However, this study also observed a non-significant change in PEF in both groups.

Results of qualitative synthesis are described in Table 3.

#### 3.2.3. Subgroup Analysis

Subgroup analyses could not be performed due to the limited number of studies [38].

## 4. Discussion

### 4.1. Summary of Evidence

This review investigated the benefits of breathing exercise on recent and persistent neck pain. Our results should be interpreted cautiously, as the certainty of evidence was low, suggesting that the true effect might be or is probably different from the estimated effect [39]. The main findings of our review can be summarized as follows: (1) Breathing exercises significantly reduced neck pain (SMD, −10.16; 95% CI: −14.82, −5.50), achieving clinically meaningful pain relief that exceeded the minimal clinically important difference (MCID) threshold of 5.5 for neck pain; breathing exercises also provided substantial improvements in neck disability (SMD, −0.80; 95% CI: −1.49, −0.11) [29]; (2) breathing exercises can promote a significant increase in lung capacity with increased FVC, [33,34], FEV1, and the FEV1/FVC ratio, with a post-training *p*-value of <0.05 when compared to the control group [40,41]; (3) concordantly, resisted respiratory muscle training was proposed in one study [35], wherein a significant increase in respiratory muscle strength after training was observed (MIP, *p* = 0.00; MEP *p=* 0.00, and MVV, *p* = 0.00) [37].

### 4.2. Breathing Exercises and Respiratory Function

Breathing exercises, particularly those based on resistive load devices, serve as a form of resistance exercise specifically aimed at strengthening the respiratory muscles, with a primary focus on the diaphragm and intercostal muscles [37]. Through consistent resistance exercises, these muscles experience muscular hypertrophy gains, enhancing both their strength and endurance, as shown in our review by higher MIP and MEP values, as well as MVV after training [37]. Additionally, increased contractile strength can boost diaphragm shortening velocity, especially when breathing exercises are associated with diaphragmatic breathing [42,43]. These adaptations support more effective respiratory mechanics, ultimately improving lung function, as demonstrated by the increased FVC, FEV1, and FEV1/FVC ratio post-training [42,43]. The hypertrophy of respiratory muscles may also influence pain modulation by improving the efficiency of breathing patterns, which can reduce the effort and strain during respiratory movements, potentially alleviating discomfort or pain. The ability to take deeper breaths allows for a greater volume of air intake into the lungs, thus promoting lung expansion and improving pulmonary function (such as FVC) [42,43]. Increased respiratory muscle function could also play a role in pain relief by improving postural stability and reducing the need for compensatory muscle recruitment, which may contribute to a reduction in musculoskeletal pain [2,3,12]. Therefore, enhanced respiratory muscle strength (i.e., MIP and MEP) post-training allows for a greater generation of negative intrathoracic pressure, thereby improving lung function efficiency, which might be linked to pain relief. Although this hypothesis aligns with the findings of this systematic review, it is also important to consider the influence of the effects that are not attributable to the specific effects, such as placebo effects, or the increased physical activity associated with the better respiratory function after breathing interventions. These may influence the therapeutic outcomes, depending on contextual effects, non-specific effects, and their mutual manifestation [44,45,46]. Although one study did not find differences in lung capacity, this may be attributed to the specific characteristics of the breathing interventions used, as two of the studies included diaphragmatic breathing exercises [33,34], which can potentialize the effects on lung volume.

### 4.3. Breathing Exercises and Neck Pain/Disability

Respiratory muscle training involves exercises with a respiratory resistance applied during the respiratory phases [16,47]. In the included study, training for expiratory muscles was provided using a positive expiratory pressure device (PEP) while subjects were asked to inhale deeply and exhale through the PEP mouthpiece forcefully [37]. Respiratory muscle training strengthens these muscles, reduces fatigue, and enhances breathing efficiency [16,47,48].

Diaphragmatic breathing stimulates the vagus nerve, reducing inflammatory cytokines, sympathetic tone, and oxidative stress, while regulating brain activity and opioid effects [8]. Additionally, improved respiratory muscle function following training can be linked to a delay in the respiratory metaboreflex [48]. This is a physiological reflex triggered by the accumulation of metabolic byproducts, such as carbon dioxide and lactate, in the respiratory muscles during intense or prolonged exercise. When these metabolites build up, they activate sensory receptors, which in turn stimulate the cardiovascular system to increase blood pressure and redistribute blood flow to support the respiratory muscles [49]. This reflex is mediated by increases on central and peripheral sympathetic nerve activity [48]. Therefore, it is reasonable that a delay on the respiratory metaboreflex after breathing exercises may contribute to chronic adaptations in basal autonomic tone, leading to lower sympathetic drive and enhanced vagal regulation [48,50].

This enhancement in vagal tone is associated with a more balanced autonomic function, which has significant implications for managing chronic pain conditions [51]. Evidence suggests that interventions aimed at reducing sympathetic overactivity can decrease muscle tension, improve blood flow, and enhance pain resilience [51,52,53]. Consequently, it is plausible that increased vagal modulation contributes to a reduction in sympathetic overactivity and systemic inflammation, factors closely linked to chronic neck pain and disability [51,52]. This autonomic adaptation may play a role in mitigating the severity of neck pain and improve functional outcomes, potentially reducing disability levels by addressing the autonomic imbalance frequently observed in individuals with chronic pain [51,52,53].

Although breathing exercise-based interventions are generally designed to enhance specific respiratory functions, such as MIP and MEP [33,34,35,36,37], research suggests an important link between reductions in respiratory strength and overactivity in accessory respiratory muscles, particularly the sternocleidomastoid and anterior scalene muscles [2,9,12,54]. This overactivity places excess strain on these muscles, potentially leading to early fatigue and impairing movement in the cervical spine and rib cage [2,9,12,54]. Consequently, such an imbalance disrupts optimal coordination among respiratory muscles and affects rib cage mechanics, increasing cervical and thoracic muscle strain, and heightening the risk of neck pain and disability [3]. In contrast, improved respiratory function through targeted training can reduce overuse of these accessory muscles, easing strain on the cervical region. This adjustment can positively influence pain levels and functional limitations in individuals with chronic neck pain [37], emphasizing the therapeutic potential of respiratory muscle training for managing neck-related pain and disability.

Compared with asymptomatic individuals, people suffering of persistent neck pain present meaningful respiratory dysfunction [2]. Chronic pain is strongly influenced by psychological factors and, intriguingly, increased respiratory dysfunction has also been observed with people presenting decreased mental and emotional health [6]. Breathing exercises were found to be effective in preventing and reducing the effect of psychological factors such as anxiety and stress [55,56]. Therefore, it is reasonable that the reduction in pain and disability observed in our review could also be determined by this effect.

### 4.4. Implication for Practice

Our results align with previous systematic reviews [2,3,7,13] on this topic, which observed an association between persistent neck pain and respiratory dysfunction, recommending integrating breathing exercises in the management of persistent neck pain. A recent review [13] suggests that clinicians should incorporate respiratory function assessments and breathing exercises into a multimodal approach for the management of neck pain may enhance outcomes and accelerate recovery [5,57]. Person-centered care advocates to provide management approaches that should be tailored to the individual patient to enhance their prognosis [58,59]. Educating patients on performing breathing exercises autonomously may empower self-efficacy and maintain improvements achieved during conservative treatment [60,61]. Therefore, breathing exercises could offer a cost-effective solution and should be considered in the management of persistent neck pain as they are easy to perform and do not require sophisticated or expensive equipment [62].

### 4.5. Strengths and Limitations

In the present study, meta-analysis for respiratory functional parameters, as well as subgroup analysis, could not be performed due to the limited number of available studies. Although there are a limited number of included studies, a meta-analysis allows for the synthesis of existing data, and provides a more precise estimate of the effect size [63]. Despite the limited data, this approach enhances the robustness of the findings and allows for a more comprehensive understanding of the effects of breathing exercises on neck pain to guide decision-making [63]. The GRADE approach provided a low certainty of the evidence, acknowledging the uncertainty associated with the findings, while still highlighting their relevance to the field [39].

We found high heterogeneity in all of the assessed outcomes, which may be attributed to the differences between the populations of the included studies, the use of clustered interventions, and their variability. As an example, two studies [33,37] involved patients over 40 years old, while two studies [35,36] included patients under 40 years old. One study [35] included only females. A difference in BMI was also found, as in three studies [33,35,37], overweight patients were included, while one study had patients in the healthy weight range [36]. In addition, breathing exercises encompass a broad range of interventions, including the type of breathing exercises (i.e., diaphragmatic breathing, balloon breathing, and respiratory muscle training), dosage, and frequency, as well as intervention duration, which ranged from 2 weeks to 8 weeks. This heterogeneity may impact the generalizability of the findings and suggests for caution to be adhered to in interpreting the results across different subgroups. Additionally, the variability in study designs and outcomes may contribute to the potential for overestimating the intervention effects. Furthermore, publication bias and limited reporting of negative results must be considered as potential factors influencing the findings. The lack of reporting on negative or neutral results in some studies may create an inflated perception of the effectiveness of breathing exercises.

### 4.6. Future Perspectives

There is a paucity in the literature investigating respiratory assessment and treatment for people suffering from neck pain [13]. No studies were found that investigated the effect of respiratory exercises on recent neck pain, evaluated their long-term effects, or assessed quality of life. Future high-quality studies, like RCTs, are needed to minimize bias and determine the true effect of breathing exercises in the long-term on both persistent and recent neck pain patients by examining the cause–effect relationships between the intervention and the outcomes [64].

## 5. Conclusions

Breathing exercises provide short-term beneficial effects in reducing pain and disability compared to other non-invasive conservative interventions for persistent neck pain with a low certainty of evidence. Breathing exercises provided statistically significant benefits for functional respiratory parameters. Future RCTs on breathing exercises interventions are needed to better understand the long-term effect on persistent and recent neck pain patients.

### Implications for Rehabilitation

- Breathing exercises can reduce pain and disability and may improve short-term pulmonary function for individuals with persistent neck pain.
- Although the mechanisms are not fully understood, an assessment of respiratory function and breathing exercises should be part of a multimodal approach to manage persistent neck pain.
- The certainty of evidence is low for all of the outcomes, preventing definitive conclusions about the effect of breathing exercises for individuals with persistent neck pain.

## Figures and Tables

**Table 1:** Characteristics of the included studies.

Study	Setting	Population		Experimental		Control		Outcomes	Follow-Up
			Design	Baseline Characteristics	Intervention	Baseline Characteristics	Intervention		
Anwar 2022 [34]	University of Lahore, Lahore, Pakistan	Non-specific chronic neck pain for more than three months	RCT	Age, y: 38.54 ± 6.72 Height, cm:158.1 ± 6.33 Weight, kg: 64.85 ± 8.15 BMI, kg/m^2^: 25.84 ± 1.51 Male/Female: 8/7	Physiotherapy and Breathing reeducation	Age, y: 38.42 ± 5.12 Heights, cm: 156.71 ± 8.26 Weight, kg: 62.57 ± 8.14 BMI, kg/m^2^: 25.37 ± 8.26 Male/Female: 6/9	Routine physical Therapy and sham-breathing exercise	Cervical muscles Endurance, Cervical muscle strength, Pulmonary functions: (FEV1, FVC, FEV1/FVC)	4th and 8th weeks
Anwar 2022 [33]	Independent Medical College Faisalabad, Faisalabad, Pakistan,	Non-specific chronic neck pain for more than three months	RCT	Age, y: 39.71 ± 5.56 Height, cm: 156.35 ± 4.64 Weight, kg: 65.15 ± 6.96 BMI, kg/m^2^: 27.01 ± 1.67	Physiotherapy and Breathing reeducation	Age, y: 39.00 ± 4.90 Height, cm: 156.44 ± 3.66 Weight, Kg: 63.86 ± 6.09 BMI, Kg/m^2^: 26.67 ± 1.65	Routine physical Therapy and sham-breathing exercise	Pain: (Vas), Cervical ROM, Disability: (NDI), Pulmonary functions: (FEV1, FVC, FEV1/FVC)	4th and 8th weeks
Balaganapathy 2022 [37]	Rita Patel Institute of Physiotherapy, Anand, Gujarat, India	Diagnosed Chronic Neck Pain for more than three months	RCT	Age, y: 42.50 ± 7.25 Height, cm: 1.67 ± 0.70 Weight, kg: 74.70 ± 16.39 BMI, kg/m^2^: 26.72 ± 6.38 Male/Female: 12/8	the respiratory muscle training and Interferential Current therapy and stretching of neck muscles	Age, y: 39.30 ± 8.19 Height, cm: 1.59 ± 0.06 Weight, kg: 64.70 ± 8.39 BMI, kg/m^2^: 25.33 ± 2.92 Male/Female: 14/6	Interferential Current therapy and stretching of neck muscles	Pain: (NPRS), Disability: (NDI), Pulmonary functions: (MIP, MEP, FVC, FEV1/FVC, PEFR, SVC, MVV)	4th week
Dareh-Deh 2022 [36]	Kharazmi University, Tehran, Iran	smartphone users with FHD and Non-Specific Chronic Neck Pain	RCT	Age, y: 23.9± 2.3 Height, cm: 177.8 ± 5.4 Weight, kg: 71.8 ± 6.0 BMI, kg/m^2^: 22.6 ± 1.1	Therapeutic routine and breathing exercise (balloon breathing)	Age, y: 24.9 ± 2.8 Height, cm: 177.0 ± 5.7 Weight, kg: 72.2 ± 4.2 BMI, kg/m^2^: 23.8 ± 1.2	Therapeutic routine: resistance and stretching exercises	Pain: (VAS), Forward head angle: (photogrammetry), MVC of specific muscles: (electromyography), respiratory patterns: (manually),	8th week
Mosallaiezadeh 2023 [35]	Tehran University of Medical Sciences, Tehran, Iran	Chronic Neck Pain for more than three months	RCT	Age, y: 27.80 ± 2.83, Height, cm: 165.14 ± 1.95 Weight, kg: 71.43 ± 6.89 BMI, kg/m^2^: 26.21 ± 2.76 15 females	Diaphragmatic Exercises and physiotherapy.	Age, y: 27 ± 2.61 Height, cm: 164.75 ± 1.8 Weight, kg: 73.35 ± 5.09 BMI, kg/m^2^: 27.05 ± 2.19 15 females	Physiotherapy (TENS strength and stretching exercises)	Pain: (VAS), Disability: (NDI), cervical active ROMs, and FHP	2nd week

Summary: Two studies were conducted in Pakistan, one in India, and two in Iran. All studies were published in English. All of the studies included patients with persistent neck pain. Different types of breathing exercise used in the experimental arm are as follows: diaphragmatic exercises, respiratory muscle training, and balloon breathing. Four articles assessed pain with VAS and NPRS, and three assessed disability with the NDI. Two studies evaluated FEV 1 with the spirometry. Three studies evaluated FVC with the spirometry. Three studies evaluated the ratio FEV1/FVC with the spirometry. Abbreviations: RCT, Randomized Controlled Trial; BMI, Body Mass Index; TENS, Transcutaneous Electrical Nerve Stimulation; NPRS, Numeric Pain Rating Scale; VAS, Visual Analogue Scale; NDI, Neck Disability Index; FEV1, Forced Expired Volume in the first second; FVC, Forced Vital Capacity; MIP, Maximal inspiratory; MEP, Pressure, Maximal Expiratory Pressure; PEFR, Peak Expiratory Flow Rate; SVC, Slow Vital Capacity; MVV, Maximal Voluntary Ventilation; ROM, Range of Motion; FHP, Forward Head Posture; cm, Centimeter; y, Years; and kg, Kilogram.

**Table 2:** Summary of treatment effects and GRADE summary of findings among trials included in the systematic review of breathing exercises for neck pain.

Primary Analyses: Breathing Exercise Compared to Routine Physiotherapy					
Analyses	Effect Estimate (95% CI)	No. of Participants	No. of RCTs	I^2^ (%)	Quality of Evidence (Reason for Downgrading)
Pain (VAS and NPRS).Follow-up: mean 8 weeks	SMD **10.16 SD lower** (14.82 lower to 5.5 lower)	178	4	98	Low (high risk of bias, high heterogeneity, wide confidence intervals, sample size)
Disability (NDI). Follow-up: mean 8 weeks	SMD **0.8 SD lower** (1.49 lower to 0.11 lower)	138	3	72	Low (high risk of bias, high heterogeneity, wide confidence intervals, nonreporting biases)

Abbreviations: I^2^, heterogeneity; GRADE, Grading of Recommendations, Assessment, Development, and Evaluation; CI, confidence interval; SMD, standardized mean difference; VAS, visual analog scale; NPRS, Numeric Pain Rating Scale; and RCT, randomized controlled trial.

**Table 3:** Qualitative synthesis of studies with outcomes excluded from meta-analysis.

Study	Experimental	Control	Outcomes and Follow-Up	Conclusions
Anwar 2022 [34]	n = 15 Physiotherapy and Breathing reeducation	n = 15 Physiotherapy and sham-breathing exercise	FEV1 FVC FEV1/FVC 8 weeks	A significant increase in FVC (*p* = 0.020) was found for breathing reeducation group at 8 weeks post-treatment. No statistically significant differences between groups improvement for FEV1 (*p* = 0.830) and FEV1/FVC (*p* = 0.602 was found at 8 weeks post-treatment.
Anwar 2022 [33]	n = 34 Physiotherapy and Breathing reeducation	n = 34 Routine physical Therapy and sham-breathing exercise	FEV1 FVC FEV1/FVC 8 weeks	A significant increase in FEV1 (*p* = 0.045), FVC (*p* < 0.001), and FEV1/FVC ratio (*p* < 0.001) in the breathing reeducation group was found at 8 weeks post-treatment.
Balaganapathy 2022 [37]	n = 20 Respiratory muscle training and Interferential Current therapy and stretching of neck muscles	n = 20 Interferential Current therapy and stretching of neck muscles	FVC FEV1/FVC MVV MIP MEP PEF 4 weeks	A significant increase in MIP (*p* = 0.00), MEP (*p* = 0.00), and MVV (*p* = 0.00) in the breathing reeducation group was found at 4 weeks post-treatment. No statistically significant differences between groups improvement for FVC (*p* = 0.80), FEV1/FVC and PEF was found at 4 weeks post-treatment.

Abbreviations: FEV1, Forced Expired Volume in the first second; FVC, Forced Vital Capacity; MIP, Maximal inspiratory; MEP, Pressure, Maximal Expiratory Pressure; MVV, Maximal Voluntary Ventilation.

## Data Availability

Data are contained within the article.

## Appendix A. Search Terms and Strategies

The search strategy for PubMed (MEDLINE), PEDro, CINAHL, Scopus, and EMBASE was established on 28 February 2024. The search terms included descriptions of neck pain and breathing exercises. Each search string was tailored for the specific database it was intended to search. It utilized both medical subject headings (MeSH) and free terms for each aspect of the PICOS framework (population, intervention, comparator, outcome, study design). MeSH terms and relevant free-text words were combined using Boolean operators (e.g., AND, OR).

**Database**	**Search Strategy**
PubMed	(“breathing exercises” [MeSH Terms] OR “respiration” [Title/Abstract] OR “deep breathing” [Title/Abstract] OR “diaphragmatic breathing” [Title/Abstract]) AND (“Neck Pain” [MeSH Terms] OR “neck pain*” [Title/Abstract] OR “cervicalgia*” [Title/Abstract] OR “cervical pain*” [Title/Abstract])
PEDro	Abstract and Title: breathing Problem: pain Body part: head or neck Method: clinical trial When Searching: match all search term (AND)
Scopus	(TITLE-ABS-KEY (“neck pain”) AND TITLE-ABS-KEY (“breathing exercise” OR “deep breathing” OR respiration))
CINAHL	neck pain AND (breathing exercises OR respiration OR deep breathing)
EMBASE	(“breathing exercises” OR “respiration” OR “deep breathing” OR “diaphragmatic breathing”) AND (“Neck Pain” OR “neck pain*” OR “cervicalgia*” OR “cervical pain*”)

## Appendix B. Included Studies

**No.**	**Author**	**Year**	**Title**	**DOI**
1	Anwar et al. [34]	2022	Effects of breathing re-education on endurance, strength of deep neck flexors and pulmonary function in patients with chronic neck pain: A randomized controlled trial.	https://doi.org/10.4102/sajp.v78i1.1611
2	Anwar et al. [33]	2022	Effects of breathing reeducation on cervical and pulmonary outcomes in patients with non specific chronic neck pain: A double blind randomized controlled trial.	https://doi.org/10.1371/journal.pone.0273471
3	Balaganapathy and Kansara [37]	2022	Respiratory Muscle Strength Training and Pulmonary Function Changes in Subjects with Chronic Neck Pain.	https://doi.org/10.1007/978-981-16-7361-0_27
4	Dareh-deh et al. [36]	2022	Therapeutic routine with respiratory exercises improves posture, muscle activity, and respiratory pattern of patients with neck pain: a randomized controlled trial.	https://doi.org/10.1038/s41598-022-08128-w
5	Mosallaiezadeh et al. [35]	2023	Effects of Combining Diaphragmatic Exercise with Physiotherapy on Chronic Neck Pain: A Randomized Clinical Trial.	https://doi.org/10.18502/jmr.v17i1.11307

## Appendix C. Excluded Studies

**No.**	**Title**	**Authors**	**Year**	**Reason for exclusion**	**Journal**
1	Immediate Effects and Acceptability of an Application- Based Stretching Exercise Incorporating Deep Slow Breathing for Neck Pain Self-management	Thongtipmak	2020	Breathing exercise were combined with other treatment and was impossible to differentiate the data	Healthcare Informatic Research
2	Breathing retraining with chest wall mobilization improves respiratory reserve and decreases hyperactivity of accessory breathing muscles during respiratory excursions: A randomized controlled trial	Sakuna	2020	Breathing exercise were combined with other treatment and was impossible to differentiate the data	Acta of Bioengineering and Biomechanics
3	Effects of static contraction and cold stimulation on cardiovascular autonomic indices, trapezius blood flow and muscle activity in chronic neck–shoulder pain	Hallman	2011	The present study does not respond to our research question	European Journal of Applied Physiology
4	Effect of physiotherapy on respiratory functions in patients with chronic neck pain	Duymaz	2019	Breathing exercise were combined with other treatment and was impossible to differentiate the data	The Annals of Clinical and Analytical Medicine
5	Biofeedback-Assisted Relaxation Training for the Aging Chronic Pain Patient	Middaugh	1991	Wrong study design and the present study does not respond to our research question	Biofeedback and Self-Regulation
6	Effect of diaphragmatic breathing, respiratory muscle stretch gymnastics and conventional physiotherapy on chest expansion, pulmonary function and pain in patients with mechanical neck pain: A single group pretest-posttest quasi-experimental pilot study	Chand	2023	breathing exercise were combined with other treatment and was impossible to differentiate the data	Journal of Bodywork & Movement Therapies
7	Respiratory muscle endurance training reduces chronic neck pain: A pilot study	Wirth	2016	Wrong study design	Journal of Back and Musculoskeletal Rehabilitation
8	Effects of breathing re-education on clinical outcomes in patients with non-specific chronic neck pain	Anwar	2022	Wrong study design	Journal of Pakistan Medical Association
9	Muscle stretching with deep and slow breathing patterns: a pilot study for therapeutic development	Wongwilaira	2018	Wrong study design	Journal of Complementary and Integrative Medicine
10	A randomized clinical trial of self-stretching with and without mindful breathing—immediate effect on pressure pain and range of motion in myofascial pain syndrome	Buranruk	2022	Wrong study design	Journal of Bodywork & Movement Therapies
11	Effect of respiratory exercises on neck pain patients: A pilot study	Mohan	2016	Wrong study design	Polish annals of medicine

## Appendix D. Template for Intervention Description and Replication (TIDieR)

	**Anwar et al., 2022 (1)**	**Anwar et al., 2022 (3)**	**Dareh Deh et al., 2022**	**Mosallaiezadeh et al., 2023**	**Balaganapathy et al., 2022**
1. BRIEF NAME	Physiotherapy and sham breathing exercises (BE) vs. Physiotherapy and Supervised breathing exercises (BE)	Breathing reeducation (BR) vs. routine physical therapy (RPT)	Routine physical therapy (RPT) with respiratory exercises (BE) vs. routine physical therapy (RPT) without respiratory exercises (BE)	Diaphragmatic exercises and physiotherapy (DEPT) vs. physiotherapy alone (PT)	Respiratory muscle training (IMT device and PEP device) and standard treatment (Interferential Current therapy and stretching of neck muscles like upper trapezius, sternocleidomastoid and scalene) vs. standard treatment
2. WHY	Primary aim: “to examine the effects of BE combined with physiotherapy on endurance and strength of deep neck flexors, and pulmonary function in patients with NSPNP.”	Primary aim: “to study effects of breathing reeducation in the treatment of patients with NSPNP”	Primary aim: “to compare the effect of RPT with and without BE on smartphone users with FHP and NSPNP”	Primary aim: “to determine the effect of combining diaphragmatic exercises with physiotherapy on pain, disability, and CAROMs and FHP in individuals with NSPNP.”	Primary aim: “to find out the changes in respiratory muscle strength and pulmonary functions in subjects with NSPNP.”
3. WHAT materials	Craniocervical Flexion Test by a pressure sensor placed behind the neck. Cervical muscle strength was measured with a handheld dynamometer (Baseline Lite 200 lb) Pulmonary function was measured with spirometry with the Spirolab4 (USA).	VAS. CROM (basic) device by Performance Attainment Associates TM(USA) NDI (Urdu Version). Straight push pad of handheld dynamometer (Baseline Lite 200lb) for strength of cervical flexors and extensors. Spirolab4 for pulmonary functions.	VAS. NDI. FHP, lateral photograph (photogrammetry of the sagittal plane). EMG device with eight channels (made by data Log Biometrics company, Canada). Manual Assessment of Respiratory Motion (MARM) for respiratory pattern assessment.	VAS (ranges from 0 to 10 cm and a higher score indicates un- bearable pain). NDI. CAROM (goniometry). FHP by lateral photograph.	NPRS. NDI. MIP, MEP with the respiratory pressure meter pulmonary functions like FVC, FEV1/FVC, PEFR, SVC and MVV. All of these parameters were measured with a spirometer (RMS Helios 702) and were recorded with RMS Helios 702 software version 3.1.85 using a laptop.
4. WHAT procedures	“Physiotherapy consisting of infrared radiation (IRR) over the cervical region in prone for 10 min followed by isometric exercises for flexors and extensors of the cervical spine in supine with a 10 s hold for each muscle group. 20 repetitions were performed. The physiotherapy was followed by sham breathing exercises for 15 min. For sham breathing exercises, each patient was instructed to lie in supine and place one hand on the chest and the other hand on the belly or navel region and breathe in their normal manner. Physiotherapy and supervised breathing exercises focusing on proper inhalation, exhalation, and chest expansion for 15 min. Each patient was instructed to place one hand on the chest and the other hand on the belly or navel region, inhale slowly through the nose for 5 s–8 s, and exhale slowly through the mouth relaxing the chest wall and the abdomen. The duration of each session was 30 min and the total treatment time for both groups was the same.” “Patients in both groups received the intervention five days a week for a consecutive eight weeks.”	“In routine physical therapy group treatment comprised of infrared radiation (IRR) and isometric exercises of the neck muscles. Patients were instructed to lie in prone position and IRR was applied for 10 min on cervical region, followed by isometric exercises for cervical muscles (flexors and extensors) in supine lying with 10 s hold and 20 repetitions. After that each patient was instructed to perform placebo breathing exercises for 15 min. It was unsupervised random shallow routine breathing.”	“Training included two parts: therapeutic routine and respiratory exercises. The therapeutic exercises contained resistance and stretching exercises (in the three stretching exercises, they used static stretching with a 30-s hold for 2-sets) for 45 to 60 min per session, specifically one session a day for three sessions a week; totally all held in eight weeks 36–40. The rest interval between movements in these exercises was 45 and 30 s for resistance and stretching exercises, respectively. Resistance exercises included: 1. Sidelying external rotation (Teres minor, infraspinatus), 2. Prone horizontal abduction with external, rotation (Middle trapezius, Lower trapezius, Rhomboids, Infraspinatus, Teres minor), 3. Y-to-I exercise (Middle trapezius, Lower trapezius, Serratus anterior): Subjects try to flex the shoulder 180 degrees while externally rotating while in the prone position with the shoulder at a 90-degree abduction, 4. Chin tuck (Longus colli, Longus capitis): Subjects bring the chin close to the chest while lying on the supine position. Stretching exercises included: 1. Static elevator scapulae stretch (elevator scapulae) exercise (Pectorals minor), 2. One-sided unilateral self-stretch exercise (Pectorals minor): Subjects stands back against the wall at a distance, and while placing one forearm on the wall, the body rotates in the opposite direction, 3. Static sternocleidomastoid stretch. In the combined group, respiratory exercises were added to the therapeutic routine above, which consisted of balloon breathing exercises performed in sessions of four sets: The subject lies in the supine position, placing the soles of his feet against the wall so that the ankle, knee, and thigh joints are at a 90-degree angle. The subject places a 3–4-inch ball between his/her knees, which he/she has to maintain through the pressure of the internal thigh muscles during the whole training period and puts his/her back on the bed through a flat pelvic tilt. Holds the right hand above the head and the left hand with the balloon. It inhales through the nose in 3–4 s and then exhales slowly into the balloon. To perform the next tail operation, place only the tongue on the roof of the mouth without biting the balloon to prevent air from escaping inside the balloon, and as each set had four complete breathing breaks, these exercises were conducted for two sessions a day and three days a week for eight weeks. All exercise was done under the supervision of a physical therapist at the pain clinic. All participants received documentation, including information on postural corrections, and improving general health.” “Control group: received a pamphlet including information on postural corrections and improving general health during the 8-week study period. No other physical therapy modalities or treatments were performed.”	“The DEPT group received diaphragmatic exercise and physiotherapy. The diaphragmatic exercise was performed in a supine position with 40° trunk flexion while holding 2.5 kg on the abdomen in the first 5 sessions and then 5 kg in the second 5 sessions. Participants performed 3 sets with 10 repetitions at a ratio of one second of inspiration to two seconds of expiration, three sets of 15 repetitions at a ratio of two seconds of inspiration to four seconds of expiration, and three sets of 20 repetitions at a ratio of three seconds of inspiration to six seconds of expiration. The rest between rests was 60 s. These exercises were performed 5 days a week in 10 sessions.” “Physiotherapy included Transcutaneous Electrical Nerve Stimulation (TENS) on the painful regions around the neck for 30 min with a TENS device (NOVIN, Model 735X).” “TENS parameters were 150 μs square pulses with a frequency of 80 Hz. The intensity of the current was adjusted to produce no contraction. Infrared (TAVANBAKHSHNOVIN, Model Single Lamp Unit) was used on the neck and back for 20 min each session. The lamp was placed at a distance from the patient’s body to create a good feeling in the person and two types of strength and stretching exercises (The strength exercise included the chin tuck head lift exercise. In the first 5 sessions, the subjects had tucked the chin and lifted the head off the table inclined at a 60° angle. When the patient held this position for 10 s, the inclination angle of the table gradually decreased by 10°, and the holding sequence was repeated for 10 s. This exercise was performed until the inclination angle of the table reached 30°. In the second 5 sessions, the participants had tucked the chin and lifted the head off the table inclined at 30° angle. Similar to the previous exercise, the inclination angle of the table progressively decreased by 10° and this exercise was performed until the inclination angle of the table reached 0°. This exercise was performed ten times in each of the four angles and Stretching Exercises (SCM and upper trapezius [UT] stretching exercises were carried out for 30 s with repetition 3 times per session while the person was sitting on a chair with both feet resting flat on the floor).” “PT group received physiotherapy alone (similar to the physiotherapy of the DEPT group). The duration of each session in the two groups was 60 to 70 min.”	“Treatment by IMT and PEP: The inspiratory muscle training was given with the subjects in a sitting position. Mouthpiece was sealed between the lips. Subjects were asked to inhale as deeply as possible through the mouthpiece. The test was repeated for four to six times, once a day for four weeks.” “The training for expiratory muscles was given using a positive expiratory pressure device. For PEP, subjects were asked to assume a sitting position. Mouthpiece was sealed between the lips. The subject was asked to inhale deeply and exhale through the PEP mouthpiece as forcefully as can. The test was repeated for four to six times, once a day for four weeks.” “Stretching and Interferential current: for stretching maneuver, subjects were asked to be in the sitting position on a chair. The stretching maneuver for upper trapezius, scalene and sternocleidomastoid muscles were given with three repetitions and 15 s hold daily for four weeks. The interferential current therapy was given to the subjects with the patient in a sitting position. The electrical current was applied to the affected part of neck region using four electrodes using two channels such that the two channels cross each other in affected area. It was given for 15 min.”
5. WHO PROVIDED	“The intervention was conducted by a senior physiotherapist with more than 10 years’ experience in musculoskeletal and cardiopulmonary physiotherapy. All the outcome measures were assessed by an independent assessor blinded to group allocation.”	“The supervision was done by an experienced physical therapist with more than ten years of experience in musculoskeletal and cardiopulmonary physical therapy.”	“The same physiotherapist and trainer supervised both active treatment groups. A PhD trained physiotherapist performed a physical therapy evaluation with 25-years of clinical experience. All exercise was done under the supervision of a physical therapist at the pain clinic.”	“The individuals were referred by physicians to the physiotherapy clinic at the School Rehabilitation. Characteristics and initial examinations were measured and recorded by an experienced therapist”	Not specified
6. HOW	Face-to-face interventions	Face-to-face interventions	Face-to-face interventions	Face-to-face interventions	Face-to-face interventions
7. WHERE	Physiotherapy department district headquarter hospital Faisalabad, Pakistan	Physiotherapy Department District Headquarter Hospital Faisalabad, Pakistan.	The Laboratory of Biomechanics and Sports Injuries Department, Kharazmi University, Tehran, Iran	Physiotherapy clinic at the School Rehabilitation, Tehran University of Medical Sciences (TUMS).	Ashok & Rita Patel Institute of Physiotherapy
8. WHEN and HOW MUCH	“The duration of each session was 30 min and the total treatment time for both groups was the same. Patients in both groups received the intervention five days a week for a consecutive eight weeks.”	“The total treatment time for both groups was the same. Patients of both groups received the intervention five days a week for consecutive 8 weeks”	“Training protocol: one session a day for three sessions a week; totally all held in eight weeks.” “Control group: received a pamphlet including information on postural corrections and improving general health during the 8-week study period. No other physical therapy modalities or treatments were performed.”	“These exercises were performed 5 days a week in 10 sessions. The duration of each session in the two groups was 60 to 70 min.”	“IMT and PEP were repeated for four to six times, once a day for four weeks.” “The stretching maneuver was given with three repetitions and 15 s hold daily for four weeks.” “The electrical current was applied to the affected part of neck region using four electrodes using two channels such that the two channels cross each other in affected area. It was given for 15 min”
9. TAILORING	N/A	N/A	N/A	N/A	N/A
10. MODIFICATIONS	NO	NO	NO	NO	NO
11. HOW WELL	NO	NO	NO	NO	NO
12. HOW WELL:	NO	NO	NO	NO	NO
Abbreviations: BE, breathing exercises; BR, breathing reeducation; RPT, routine physical therapy; DEPT, diaphragmatic exercises and physiotherapy; PT, physiotherapy; IMT, inspiratory muscle training; PEP, positive expiratory pressure; CAROM, active range of motions of cervical; FHP, forward head posture; NSPNP, non-specific persistent neck pain; VAS, visual analogic scale; NDI, neck disability index; CROM, cervical range of motion; EMG, electromyography; MARM, manual assessment of respiratory motion; NPRS, numeric pain rating scale; MIP, maximal inspiratory pressure; MEP, maximal expiratory pressure; FVC, forced vital capacity; FEV, forced expiratory volume; PEFR, peak expiratory flow rate; SVC, slow vital capacity; MVV, maximal voluntary ventilation; IRR, infrared radiation; TENS, transcutaneous electrical nerve stimulation; SCM, sternocleidomastoid; UT, upper trapezius; and PT, physiotherapy.					
